# Genome-wide survey and expression analysis of the OSCA gene family in rice

**DOI:** 10.1186/s12870-015-0653-8

**Published:** 2015-10-26

**Authors:** Yunshuang Li, Fang Yuan, Zhaohong Wen, Yihao Li, Fang Wang, Tao Zhu, Wenqing Zhuo, Xi Jin, Yingdian Wang, Heping Zhao, Zhen-Ming Pei, Shengcheng Han

**Affiliations:** Beijing Key Laboratory of Gene Resource and Molecular Development, College of Life Sciences, Beijing Normal University, Beijing, 100875 China; Department of Biology, Duke University, Durham, NC 27708 USA

**Keywords:** OSCA, DUF221 domain, Phylogenetic relationships, Expression profile, Osmotic stress, *Oryza*

## Abstract

**Background:**

Reception of and response to exogenous and endogenous osmotic changes is important to sustain plant growth and development, as well as reproductive formation. Hyperosmolality-gated calcium-permeable channels (OSCA) were first characterised as an osmosensor in *Arabidopsis* and are involved in the perception of extracellular changes to trigger hyperosmolality-induced [Ca^2+^]_i_ increases (OICI). To explore the potential biological functions of OSCAs in rice, we performed a bioinformatics and expression analysis of the *OsOSCA* gene family.

**Results:**

A total of 11 *OsOSCA* genes were identified from the genome database of *Oryza sativa* L. *Japonica*. Based on their sequence composition and phylogenetic relationship, the OsOSCA family was classified into four clades. Gene and protein structure analysis indicated that the 11 OsOSCAs shared similar structures with their homologs in *Oryza sativa* L. ssp. *Indica*, *Oryza glaberrima*, and *Oryza brachyantha*. Multiple sequence alignment analysis revealed a conserved DUF221 domain in these members, in which the first three TMs were conserved, while the others were not. The expression profiles of *OsOSCA* genes were analysed at different stages of vegetative growth, reproductive development, and under osmotic-associated abiotic stresses. We found that four and six OsOSCA genes showed a clear correlation between the expression profile and osmotic changes during caryopsis development and seed imbibition, respectively. Orchestrated transcription of three *OsOSCAs* was strongly associated with the circadian clock. Moreover, osmotic-related abiotic stress differentially induced the expression of 10 genes.

**Conclusion:**

The entire *OSCA* family is characterised by the presence of a conserved DUF221 domain, which functions as an osmotic-sensing calcium channel. The phylogenetic tree of *OSCA* genes showed that two subspecies of cultivated rice, *Oryza sativa* L. ssp. *Japonica* and *Oryza sativa* L. ssp. *Indica*, are more closely related than wild rice *Oryza glaberrima*, while *Oryza brachyantha* was less closely related. OsOSCA expression is organ- and tissue-specific and regulated by different osmotic-related abiotic stresses in rice. These findings will facilitate further research in this gene family and provide potential target genes for generation of genetically modified osmotic-stress-resistant plants.

**Electronic supplementary material:**

The online version of this article (doi:10.1186/s12870-015-0653-8) contains supplementary material, which is available to authorized users.

## Background

Drought and salt stress are major abiotic constraints affecting plant growth worldwide. The first phase common to drought and salt stress is osmotic stress [1]. Because of their sessile lifestyle, plants have developed mechanisms to avoid or cope with the consequences of water stress. Previous studies showed that plants have developed different signal transduction pathways and gene expression regulation mechanisms to perceive and respond to water deficiency [2–4]. The mechanism of the response to drought included both abscisic acid (ABA)-independent and ABA-dependent signalling cascade pathways, as well as the expression of drought-related genes, such as DREB and NAC [5–7]. The ABA-responsive element (ABRE) and its binding transcription factors are involved in ABA-dependent gene expression. Similarly, the dehydration-responsive element (DRE) and its binding protein 2 transcription factors play pivotal roles in ABA-independent gene expression in response to osmotic stress [7]. ABA synthesised after water deficit potently inhibits stomatal opening and promotes stomatal closure to prevent water loss. In addition, ABA-activated gene expression is associated with plant adaption to drought, involving genes such as RD22, RD29A, KIN1, and KIN2 [8]. However, the mechanism underlying the early response to osmotic stress in plants remains undiscovered.

The early events of plant adaptation to drought stress include perception of osmotic changes and consequent stress signal transduction cascades, leading to the activation of various physiological and metabolic responses, including stress responsive gene expression. A total of 16 cDNAs of the early response to dehydration (ERD) genes were isolated from Arabidopsis after treatment with dehydration for 1 h [9]. ERD4 encodes a protein containing a highly conserved DUF221 domain (domain of unknown function 221), which is common to various species [10–12]. On the other hand, osmotic stress and various other stimuli trigger increases in the cytosolic/intracellular free calcium concentration ([Ca^2+^]_i_) in plants [13, 14]. The hyperosmolality-induced [Ca^2+^]_i_ increase (OICI) occurs within 5 s, which may be the earliest detectable event in plants [13]. Blocking OICI disrupts drought and ABA-induced gene expression, suggesting that the precise regulation of OICI is crucial for activation of many signal transduction pathways triggered by external stimulation; this process is important in understanding plant sensing of external osmotic stress and other stimulations. Previous studies showed that osmotic/mechanical stimuli-gated Ca^2+^-permeable channels serve as osmosensors in bacteria and animals [15, 16], which indicated that OICI in plants is mediated by specific calcium permeable channels that function as osmosensors.

Using a calcium-imaging-based unbiased forward genetic screening strategy, Yuan et al. isolated several *Arabidopsis* mutants (*osca1*) that showed low OICI, and further characterised OSCA1 as a previously unknown hyperosmolality-gated calcium-permeable channel, suggesting that OSCA1 may be an osmosensor in *Arabidopsis* [17]. *OSCA1* belongs to a gene family with 15 members in *Arabidopsis*, and homologues are found in other plant species and throughout eukaryotes. In this family, *OSCA3.1* encoded an ERD4 protein [9]. Yuan et al. also reported that OSCA3.1-knockout mutants displayed normal OICIs, suggesting that OSCA3.1 may differ from OSCA1, reminiscent of the diverse functions of TRPs (transient receptor potential channels) in animals [17].

In the present study, we characterised OSCA family members in four species of the *Oryza* genus in silico and analysed the phylogenetic relationships among these OSCAs, as well as their expression profiles in various organs/tissues and under different osmotic-related abiotic stresses. These results can be used for functional validation studies of the rice *OSCA* genes and increase our understanding of the roles of plant OSCAs.

## Results

### Identification of *OSCA* genes

To explore the entire *OSCA* gene family in rice, we used the sequence of 15 AtOSCAs to search against the *Oryza sativa* L. ssp. *Japonica* genome in RGAP (Rice Genome Annotation Project) and the genome of *Oryza sativa* L. ssp. *Indica*, *Oryza glaberrima*, and *Oryza brachyantha* from the Ensembl Genomes database at the E-value of 1e-10. The presence of conserved DUF221 domain in their protein structure is the excusive criterion to confirm the OSCAs with The SMART program (The Simple Modular Architecture Research Tool). By removing sequence redundancies and alternative splice forms of the same gene, we identified 11 putative *OSCA* genes and named them *OsOSCA1.1* to *OsOSCA4.1*, in accordance with *Arabidopsis* orthologues (Additional file 1: Table S1). *OsOSCA3.1* was *OsERD4*, as reported previously [10]. Next, we identified 11 *OsIOSCAs*, 12 *OgOSCAs*, and 11 *ObOSCAs* in *Oryza sativa* L. ssp. *Indica*, *Oryza glaberrima*, and *Oryza brachyantha*, respectively (Additional file 1: Table S1). There are two orthologues of *OSCA4.1* in *Oryza glaberrima*, named OgOSCA4.1_1 and OgOSCA4.1_2, which was indicative of gene duplication after *Oryza glaberrima* had split from the rice species.

### Phylogenetic relationship and gene structure of rice *OSCAs*

**Fig. 1:**
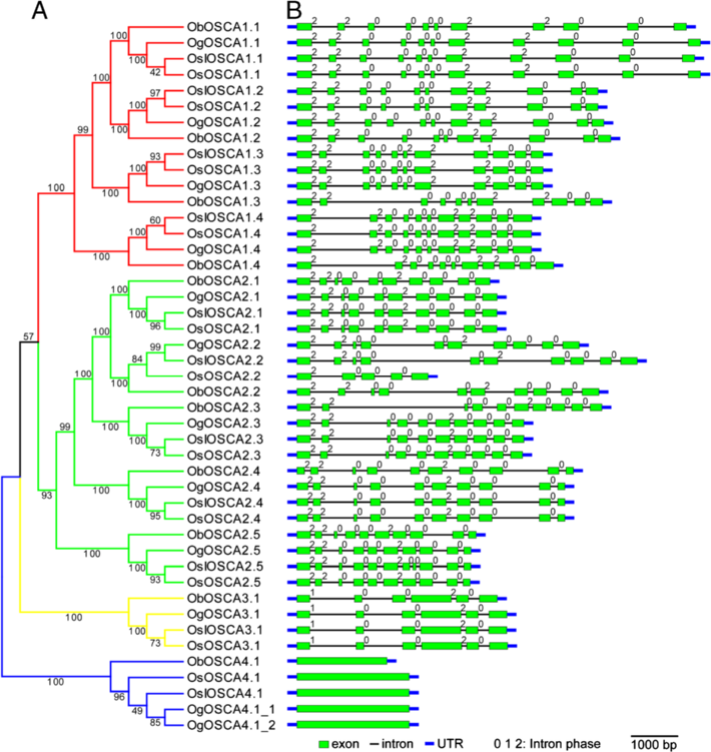
Phylogenetic relationships and gene structures of *OSCAs* in *O. sativa* L. ssp. *Japonica*, *O. sativa* L. ssp. *Indica*, *O. glaberrima*, and *O. brachyantha*. **a**. A phylogenetic tree of *OSCAs* was constructed from a complete alignment of 45 *OSCA* CDS sequences using the neighbour-joining method with bootstrapping analysis (1,000 reiterations). Bootstrap values are indicated at each node. The branches of different clades are indicated by different colours. **b**. Gene structures of *OSCAs* were identified using the Gene Structure Display Server. The horizontal black lines, the green boxes, the thick blue lines, and the numbers in the top right corner of the green boxes indicate the position of introns, the position of exons, the position of UTR (untranslated regions), and the intron phase, respectively. The length of the horizontal black lines and the green boxes represent the relative lengths of corresponding introns and exons within individual protein sequences. The scale bar at the bottom right represents a 1,000-bp length of CDS

To compare the evolutionary relationship of OSCAs among four rice species, a phylogenetic tree was generated using the CDS (Sequence coding for amino acids in protein) of 45 OSCAs. We found that members of the OSCA family were separated into four distinct clades, designated I, II, III, and IV (Fig. 1a). Clade I included four members—OSCA1.1, OSCA1.2, OSCA1.3, and OSCA1.4; Clade II contained five members—OSCA2.1, OSCA2.2, OSCA2.3, OSCA2.4, and OSCA2.5; Clades III and IV contained only OSCA3.1 and OSCA4.1, respectively. The *OSCA* gene phylogenetic tree revealed that two subspecies of cultivated rice, *Oryza sativa* L. ssp. *Japonica* and *Oryza sativa* L. ssp. *Indica*, were more closely related than wild rice *Oryza glaberrima*, while *Oryza brachyantha* was less closely related. Excluding clade IV, which contained five orthologue members, all other clades contained four orthologue, suggesting that the *OSCA* gene had duplicated, resulting in different members of the family in the ancestral species before speciation.

Previous studies have shown that the exon/intron diversification among gene family members plays an important role in the evolution of multiple gene families through three main mechanisms: exon/intron gain/loss, exonisation/pseudoexonisation, and insertion/deletion [18]. The numbers and positions of exons and introns in OSCAs were determined by comparing full-length cDNA sequences and the corresponding genomic DNA sequences of each *OsOSCA* gene using Gene Structure Display Server 2.0 (http://gsds.cbi.pku.edu.cn/). We found that all *OSCA* genes contained multiple exons with the exception of *OSCA4.1*. Moreover, in the same clade in the phylogenetic tree, most members shared almost identical intron/exon structures and intron phases (Fig. 1b). This finding further validated the nomenclature proposed by our phylogenetic analysis, and the main structural characteristics in the gene and protein sequence of OSCAs were formed prior to the split between wild and cultivated rice. However, further studies are required to elucidate the specifics of a functional divergence among the *OSCA* genes.

### The conserved domain of OSCAs

We used the SMART to confirm the structural characterisation of the OSCAs and found that most OsOSCAs contained four main modular architectures, the transmembrane helices (TM) region, the low-complexity region, a coiled-coil region, and the DUF221 domain, although the number of amino acids in the *Oryza* genus varied from 481 to 812 (Additional file 5: Figure S1). The TM in OSCAs were predicted using TMHMM Server v. 2.0. We found that different OSCA members contained 6–10 TMs, and the same OSCA orthologue in the four rice species contained the same number of TMs with three exceptions. OsOSCA2.2 and ObOSCA4.1 had three TMs fewer than their orthologues, which suggested that a deletion event occurred in the genomes of *OsOSCA2.2* and *ObOSCA4.1*; OSCA2.5 had also two TMs fewer in two subspecies of *Oryza sativa* L. than its orthologues in *Oryza glaberrima* and *Oryza brachyantha*. However, there is no deletion in the genomes of *OsOSCA2.5* and *OsIOSCA2.5* (Additional file 1: Table S1 and Additional file 5: Figure S1).

**Fig. 2:**
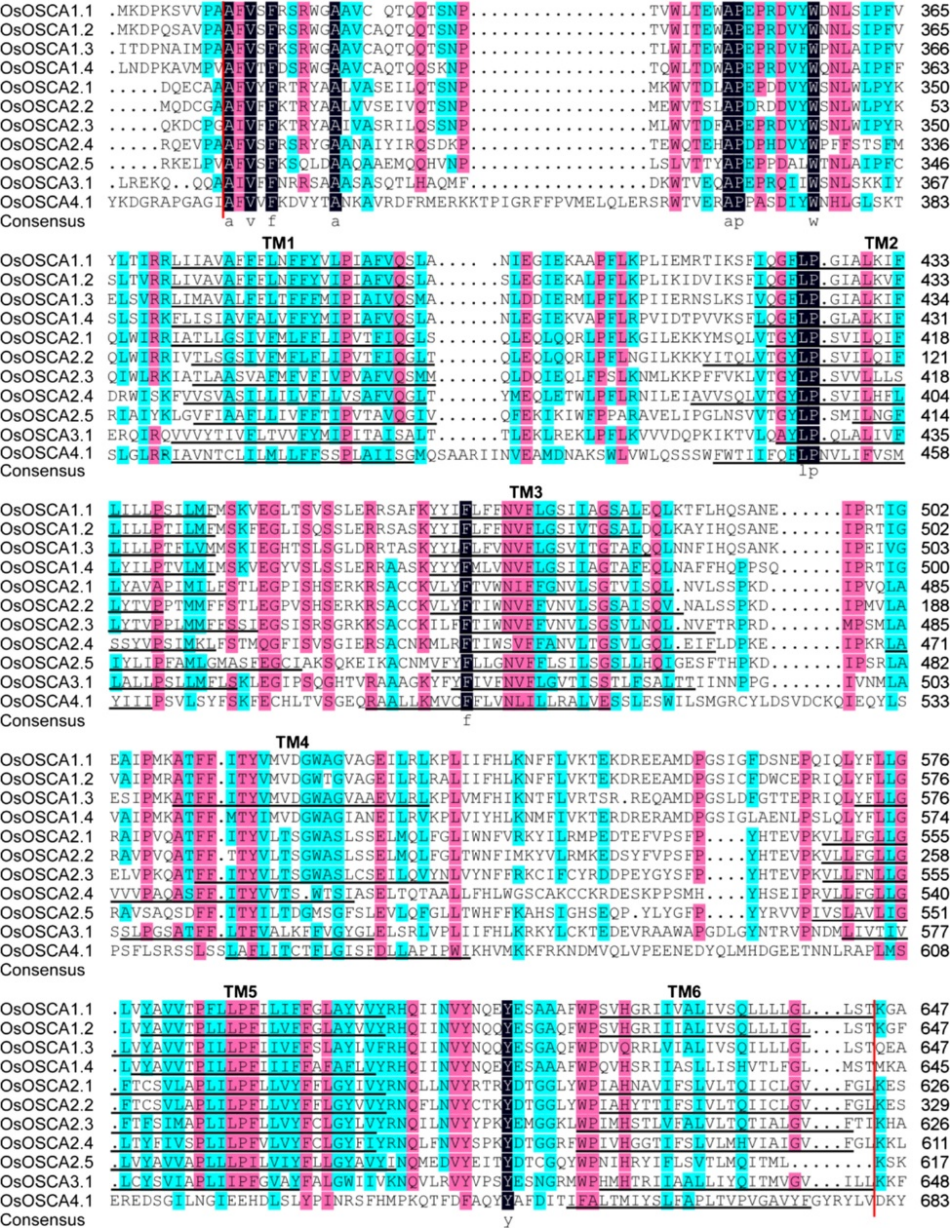
Multiple sequence alignment and transmembrane region of the DUF221 conserved region in OsOSCAs. Multiple sequence alignments of OSCAs were performed using DNAman and the transmembrane region of the DUF221 conserved region was predicted using TMHMM. The region between two vertical red lines represents the DUF221 conserved region. Identical (100 %), conserved (75–99 %), and blocks (50–74 %) of similar amino acid residues are shaded in dark navy, pink, and cyan, respectively. Identical or similar amino acid residues are shown in lowercase abbreviations at the bottom of the corresponding rows. The transmembrane regions are marked by black lines and called TM1-TM6

The entire *OSCA* family is characterised by the presence of a conserved DUF221 domain, which functions as an osmotic-sensing calcium channel [17]. According to InterPro (http://www.ebi.ac.uk/interpro/) Pfam, DUF221 represents the seven transmembrane domain region of calcium-dependent channel and is homologous to domains in anoctamin/TMEM16 channels, which are calcium-activated chloride channel (CaCC) components [19], and salt taste chemosensation transmembrane channel-like (TMC) proteins in C. elegans [20] or mechanosensitive TMCs in hair cells of the mammalian inner ear [21]. Multiple sequence alignment was performed to clarify the characteristics of DUF221 in 11 OsOSCAs (Fig. 2). In general, the core region of DUF221 contained four to six TM regions; TM1-TM3 were highly conserved in all OsOSCAs, while TM4-TM6 were not. We also identified 11 conserved amino acid residues in the DUF221 region of OsOSCAs, A319, V321, F323, A329, A349, P350, W357, L425, P426, F467, and Y613 of OsOSCA1.1, which could be associated with the channel characteristics of OSCAs.

### Expression analysis of *OsOSCA* in various organs

**Fig. 3:**
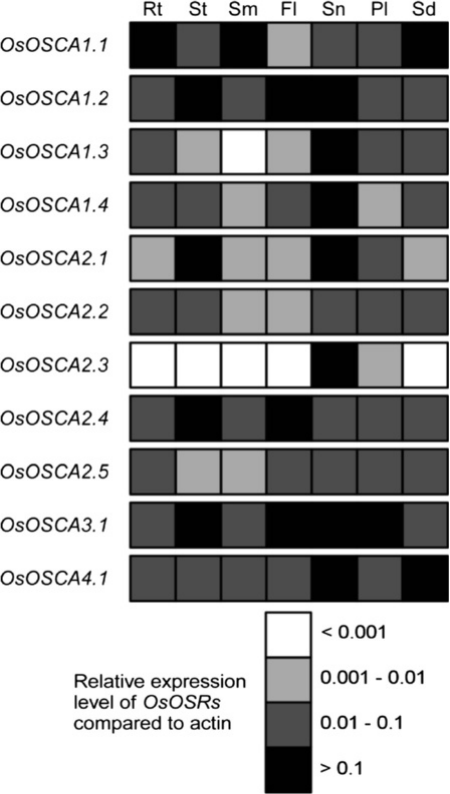
Schematic representation of organ-specific expression of the *OsOSCA* genes. The expression levels of 11 *OsOSCA*s were monitored using qRT-PCR. The values were normalised to the control gene (*actin*) and represented using a colour scale to facilitate visualisation. The letters at the top indicate the selected tissues and organs: 30-day-old root (Rt), 30-day-old shoot (St), mature stem (St), mature flag leaf (Fl), stamen (Sn), pistil (Pi), and mature seed (Sd). The colours white, light grey, dark grey, and black represent the multiple ranges of *OsOSCAs* mRNA expression levels, which were <0.001, 0.001–0.01, 0.01–0.1, and >0.1 compared with *actin*, respectively. The values represent the average of three independent biological replicates

To unveil the potential function of OsOSCAs in rice, the expression profiles of *OsOSCA* genes in various tissues and organs were first determined using qRT-PCR (real-time reverse transcription-PCR) and represented in grey scale to facilitate visualisation: 30-day-old root (Rt); 30-day-old shoot (St); mature stem (Sm); mature flag leaf (Fl); stamen (Sn); pistil (Pi); and mature seed (Sd). The 11 *OsOSCA* genes showed tissue-specific expression patterns (Fig. 3). Five genes, *OsOSCA1.1*, *OsOSCA1.2*, *OsOSCA2.4*, *OsOSCA3.1*, and *OsOSCA4.1*, were highly expressed in all tissues tested, which was indicative of a universal role of these OsOSCAs in osmotic-sensing processes throughout the plant. *OsOSCA2.2* and *OsOSCA2.5* showed medium transcript abundances in all tissues tested. *OsOSCA2.3* was detected only in the stamen, indicative of a specific function therein. *OsOSCA1.3* and *OsOSCA1.4* had relatively higher transcript abundance in the stamen and low transcript abundance in other tissues. *OsOSCA2.1* had high transcript abundance in the shoot and stamen, but low levels in other tissues. These results indicated that the *OsOSCA* genes were involved in various physiological and developmental processes in rice.

### Expression of *OsOSCAs* during caryopsis development

**Fig. 4:**
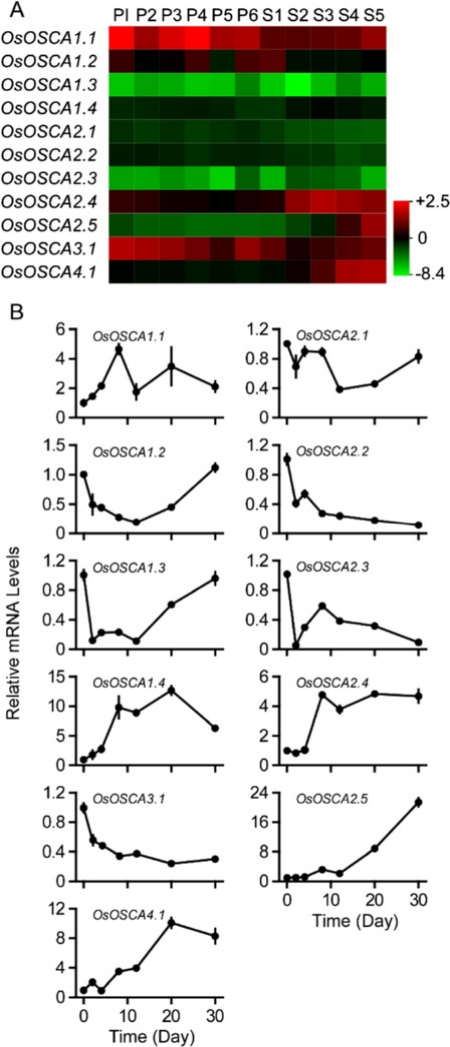
Expression profiles of *OsOSCA* genes during panicle and caryopsis development. **a**. The microarray data sets (GSE6893) of *OsOSCA* gene expression in organs at various developmental stages were reanalysed (Additional file 3: Table S3). The average log signal values of *OsOSCA* genes are presented in the form of a heat map. The colour key represents average log2 expression values of *OsOSCA* genes. The samples are indicated at the top of each lane. The following stages of panicle development are indicated as follows: P1, 0–3 cm; P2, 3–5 cm; P3, 5–10 cm; P4, 10–15 cm; P5, 15–22 cm; and P6, 22–30 cm. The following stages of caryopsis development are indicated as follows: S1, 0–2 dap (day after pollination); S2, 3–4 dap; S3, 5–10 dap; S4, 11–20 dap; and S5, 21–29 dap. A colour scale representing the average log signal values is shown on the right. **b**. The expression levels of *OsOSCA genes* during caryopsis development were monitored using qRT-PCR. Samples were collected at 0, 2, 4, 8, 12, 20, and 30 dap. The relative water content in corresponding stages is shown in Additional file 6: Figure S2. The relative mRNA levels of individual genes were normalised to that of *actin*. Error bars indicate the standard deviations (SD) of three biological replicates

To explore the transcriptional expression of *OsOSCAs* during caryopsis development after pollination, we extracted expression data of *OsOSCAs* from a published microarray database (http://signal.salk.edu/cgi-bin/RiceGE, GSE6893) and re-analysed their expression levels during various rice reproductive developmental stages, including panicles at different stages (P1–P6) and developmental seeds after pollination (S1–S5). At least one probe for each *OsOSCA* was present on the rice whole genome Affymetrix array platform (GPL2025). The 11 OsOSCA genes were divided into three subgroups, i-iii, according to their similar expression patterns (Additional file 3: Table S3 and Fig. 4a). *OsOSCA1.1*, *OsOSCA2.4*, and *OsOSCA3.1* in subgroup i were expressed with high abundance in all reproductive developmental stages of rice. Subgroup ii contained three genes, *OsOSCA1.3*, *OsOSCA2.3*, and *OsOSCA2.5*, expressed with very low abundance in almost all tissues examined. Subgroup iii included the remaining five *OsOSCAs* and showed medium abundance in all organs. More interestingly, several OsOSCAs, including *OsOSCA1.4*, *OsOSCA2.4*, *OsOSCA2.5*, and *OsOSCA4.1*, showed gradually increased expression levels during seed development, which was confirmed by qRT-PCR analysis of the expression levels of *OsOSCA* genes during caryopsis development (Fig. 4b). However, we found that the transcript levels of *OsOSCA1.1*, *OsOSCA1.2*, and *OsOSCA1.3* were increased in the developing caryopsis from the middle stage of caryopsis development (8 days after pollination) to the last stage (30 days after pollination). A decrease in *OsOSCA2.2* and *OsOSCA3.1* transcript levels was detected in the caryopsis from the earliest to the last stage of caryopsis development. OsOSCA2.3 transcript levels were higher in the caryopsis during the earliest and middle stages of development, while the expression of OsOSCA2.1 was unchanged during caryopsis development (Fig. 4b). We used the relative water content, which showed gradually decreased, as a control for caryopsis development (Additional file 6: Figure S2).

### Expression of *OsOSCAs* in the progress of rice seed imbibition

**Fig. 5:**
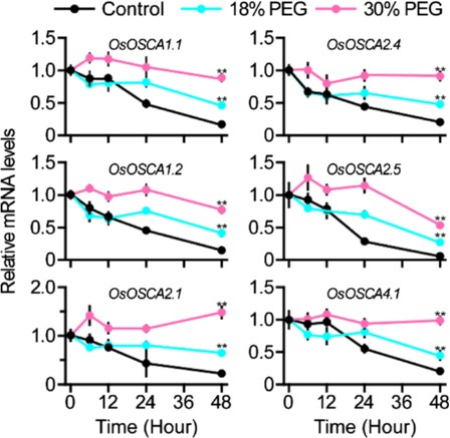
Expression analysis of *OsOSCAs* during PEG-treated seed imbibition. The expression levels of 11 *OsOSCA*s were monitored using qRT-PCR. The PEG concentration (m/v) values are represented using the colour scale shown at the top. Samples were collected after 0, 1, 6, 12, 24, 48, and 72 h of treatment with the corresponding concentration of PEG (shown on the bottom). Water content in the samples is shown in Additional file 7: Figure S3. The relative mRNA levels of individual genes were normalised to that of rice *actin*. Error bars indicate the SD of three biological replicates. The star indicates statistically significant differences (*p* < 0.05) according to Tukey’s multiple range test

Imbibition is the first and essential phase for seed germination. Water content gradually increases in seeds during this period, leading to a less negative water potential. Thus, it was important to explore whether the expression of *OsOSCAs* corresponded to osmosis variation during seed imbibition. We found that the expression levels of OsOSCA1.1, OsOSCA1.2, OsOSCA2.1, OsOSCA2.4, OsOSCA2.5, and OsOSCA4.1 were decreased during seed imbibition, from the start until 20 h, while the water content increased (Fig. 5 and Additional file 7: Figure S3). Furthermore, the lower OsOSCA transcription levels were in accordance with the increased water content during seed imbibition in 18 % and 30 % PEG (polyethylene glycol) 6000 solutions, respectively. Thus, the transcription of most *OsOSCA* genes was correlated with the water potential in imbibed seeds, which indicated that OsOSCAs play an important role in sensing and/or responding to osmotic changes to regulate seed germination.

### Orchestrated transcription of several *OsOSCAs* by the circadian clock

**Fig. 6:**
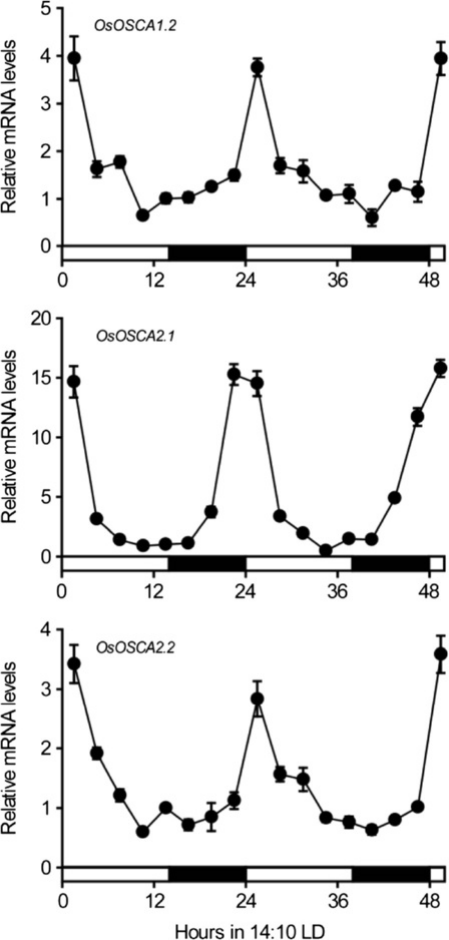
Circadian rhythmic expression of several *OsOSCAs*. Expression levels of *OsOSCA1.2*, *OsOSCA2.1*, and *OsOSCA2.2* in shoots of four-leaf stage ZH11 seedlings under 14-h light (24 °C)/10-h dark (20 °C) photoperiod conditions were analysed using quantitative real-time RT-PCR. Samples were collected every 3 h. Data were normalised against *actin* expression. The expression of marker gene is shown in Additional file 8: Figure S4. Error bars indicate the SD of three biological replicates

Water in plants is transported primarily from the root to the shoot through the transpiration stream driven by evaporation. The transpiration rate is governed by stomatal conductance, which displays diurnal oscillations [22, 23]. Thus, the water potential in the stomatal apoplast is synchronised to stomatal conductance oscillations, which may determine the circadian expression of *OsOSCAs*. To test this hypothesis, we analysed the expression profiles of all OsOSCAs in the shoots of four-leaf-stage rice seedlings under 14-h light (24 °C)/10-h dark (20 °C) conditions, and found that *OsOSCA1.2*, *OsOSCA2.1*, and *OsOSCA2.2* were subjected to a circadian rhythm at the transcriptional level (Fig. 6). During the day, stomata opening results in water loss via transpiration and higher water potential in the apoplast, which may gradually decrease the expression of *OsOSCA1.2*, *OsOSCA2.1*, and *OsOSCA2.2*. Conversely, stomata closing at night will trigger the transcription of these three *OsOSCAs*, which will peak following the dark to light transition. Except those three *OsOSCAs*, the expression of others was independent of the circadian rhythm. We used *OsLHY*, which exhibited robust rhythmic expression under diurnal conditions, as a positive control in this experiment (Additional file 8: Figure S4) [24].

### Expression profiles of *OsOSCAs* under osmotic-related abiotic stresses

**Fig. 7:**
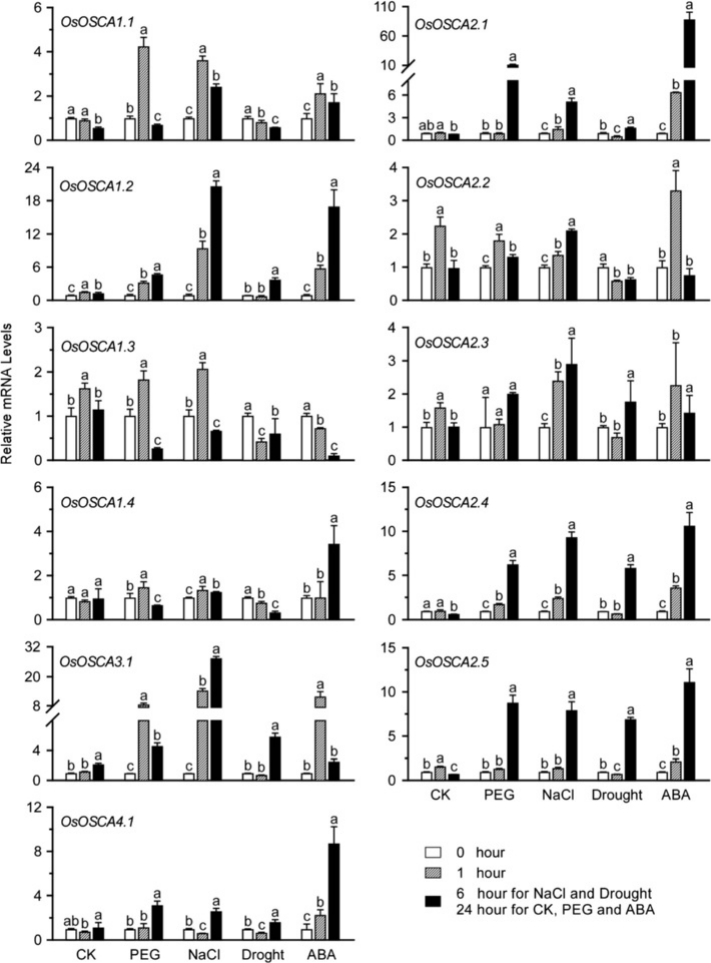
Expression profiles of *OsOSCA* genes under different abiotic stresses. The relative expression levels of *OsOSCAs* were determined using quantitative real-time RT-PCR in the roots (except for drought stress in the shoot) of four-true-leaf-stage seedlings treated with 20 % PEG 6000, 150 mM NaCl, or 100 μm ABA, compared with the control. The mRNA levels were normalised to that of *actin*. The white and grey bars represent 0 and 1 h after treatment, respectively. The dark bars represent 6 h after treatment with NaCl or Drought, and 24 h after treatment with CK, PEG, or ABA. The expression levels of marker genes are shown in Additional file 9: Figure S5. Error bars indicate the SD of three biological replicates. Different letters (a-c) indicate statistically significant differences (*p* < 0.05) according to Tukey’s multiple range test

To determine whether the expression of *OsOSCAs* is responsive to osmotic-related abiotic stress, qRT-PCR analysis of the *OsOSCAs* at the four-leaf stage in rice was performed under different stress treatments: PEG 6000 (20 %), NaCl (150 mM), drought, and ABA (100 mM). We found that nine *OsOSCA* genes were down- or upregulated (<0.5 or > 2) in at least one of the stress conditions examined as compared with the control, except for OsOSCA2.2 and *OsOSCA2.3* (Fig. 7). In detail, the expression of five genes, *OsOSCA1.2*, *OsOSCA2.1*, *OsOSCA2.4*, *OsOSCA2.5*, and *OsOSCA3.1*, were upregulated by all four kinds of treatment. *OsOSCA4.1* was upregulated by PEG and salt stress as well as ABA treatment, while *OsOSCA1.1* was upregulated by PEG and salt stress. We also found that OsOSCA1.4 was specifically downregulated by drought stress and upregulated by ABA treatment. *OsOSCA1.3* was downregulated after PEG stress and ABA treatment. The expression of marker genes, *PER24P* for PEG treatment [25], *DSM2* for salt [26], *OsP5C* for drought [27], *ABI5* for ABA [28], is shown in Additional file 9: Figure S5. We also investigated the expression of three housekeeping gene: *actin* (LOC_Os03g61970.1), *eEF1a* (LOC_Os03g08020) and *UBQ5* (LOC_Os01g22490) under different abiotic stresses and calculated the Gene expression stability values (M) of these three genes, which was 0.753 for *actin*, 0.841 for *eEF1a* and 1.069 for *UBQ5*, respectively (Additional file 4: Table S4). The M value of three genes is below the threshold value of 1.5, which showed that *actin* gene is suitable for using as the internal controls to normalize the expression of OSCA genes in rice. These results indicated that OsOSCAs might be involved in osmotic-related signalling pathways and play pivotal roles in the responses to various abiotic stresses in rice.

## Discussion

During their life cycle, plants encounter a variety of exogenous and endogenous osmotic changes and have developed various strategies to sense, respond, and adapt to these stresses. Exogenous osmotic stress includes drought, salt, temperature and the water potential in the stomatal apoplast, which is regulated by stomatal conductance. Endogenous osmotic stimuli are caused by material accumulation or consumption, such as caryopsis development, seed maturation, and seed imbibition during germination. In recent studies, OSCA was identified as an osmosensor mediating hyperosmolarity-induced cytosolic calcium increases (OICI) in *Arabidopsis*, which increased our understanding of the molecular mechanisms underlying sensing of osmotic stresses by plants [17, 29].

*Oryza* (23 species; 10 genome types) contains the world’s most important food crop, rice, which has diversified across a broad ecological range, from deep water to upland, including seasonally dry habitats. This diversification occurred within a narrow evolutionary time scale (~15 million years) due to several closely spaced speciation events, constituting an almost stepwise historical genomic record [30, 31]. Therefore, studying the phylogenetic relationship of *OSCAs* in four *Oryza* species and the expression levels of *OsOSCA* family genes in various tissue/organs, developmental stages, and under various abiotic stresses will facilitate further research on this gene family and provide potential target genes for generation of genetically modified osmotic-stress-resistant plants.

Based on the phylogenetic tree, we found that the *OSCA* genes from two *Oryza sativa* subspecies, with the exception of *OSCA2.2*, were clustered more closely with their orthologues from *Oryza glaberrima* than those from *Oryza brachyantha*, which indicated that *Oryza brachyantha* and *Oryza glaberrima* split long before the separation of cultivated and wild rice. Furthermore, the subspecies of cultivated rice, *Oryza sativa* L. ssp. *Japonica* and *Oryza sativa* L. ssp. *Indica*, have the closest relationship, which further supported the evolutionary origins in diploid *Oryza*. With the same AA genome species as *Oryza sativa*, the wild rice *Oryza glaberrima* is more closely than *Oryza brachyantha* because it is an FF genome species [30]. *OsOSCA2.2* in *Oryza sativa* L. ssp. *Japonica* lacks the first five exons compared with its orthologues in *Oryza sativa* L. ssp. *Indica, Oryza brachyantha*, and *Oryza glaberrima*, which accounts for the predicted protein structure of OsOSCA2.2 lacking the first three TM regions. In addition, *OSCA4.1* contains a single exon and *ObOSCA4.1* is shorter than its three homologues. This leads to the absence of the first three TM regions in *ObOSCA4.1*, which are present in the homologous proteins in the other three rice species. These results suggested that *OSCA4.1* is the most conserved member of the *OSCA* family, and that deletions in *OsOSCA2.2* and *ObOSCA4.1* occurred independently during rice evolution. Furthermore, we predicted that the first three TM regions may not be essential for the basic ion channel activity of OSCAs, but essential for osmosensor specificity.

In this study, we found that *OsOSCA* genes were expressed in tissue-specific patterns, indicative of a specific role for each member of the OsOSCA family in sensing various osmotic-related stresses by different tissues/organs. In addition, it was well known that osmotic conditions appear to control seed development in many plant species [32]. During caryopsis development and seed maturation after fertilisation, material accumulation and decreasing water content result in an increasing osmotic potential in endosperm cells, which may regulate the transcriptional expression of *OSCAs*. This study demonstrated that the transcription of *OsOSCA1.2, OsOSCA1.3, and OsOSCA2.5* was in accordance with increased endogenous osmotic changes during rice caryopsis development. In contrast, osmotic potential was decreased during seed imbibition, which may lower the expression of *OsOSCA1.1, OsOSCA1.2, OsOSCA2.1, OsOSCA2.4, OsOSCA2.5, and OsOSCA4.1*. These results suggest that *OSCAs* play important roles during caryopsis development and seed imbibition.

In plants, circadian rhythms control stomatal conductance, transpiration, and relative water content around the guard cells, which regulates osmotic changes in the leaf [23]. Previously, we showed that Ca^2+^-sensing receptor (CAS) mediated the external Ca^2+^ ([Ca^2+^]_o_)-induced [Ca^2+^]_i_ increase in guard cells and [Ca^2+^]_o_-induced stomatal closure [33]. We further showed that [Ca^2+^]_i_ oscillations were synchronised to [Ca^2+^]_o_ oscillations through the CAS/IP3 pathway in *Arabidopsis thaliana* [34]. In this study, we showed that the expression of *OsOSCA1.2*, *OsOSCA2.1*, *and OsOSCA2.2* was orchestrated by the circadian clock, suggestive of their potential roles in sensing and responding to extracellular osmotic changes caused by circadian rhythms. Previous extensive research showed that plants respond and adapt to drought and high-salinity stresses by inducing the expression of a number of genes [1, 35]. PEG, NaCl, and drought stress are often interconnected and may induce similar cellular damage [36], as osmotic stress is the first and primary component of salt and drought stress upon exposure of plants to high NaCl concentrations and water-deficient environments [1]. And ABA, a key plant stress-signalling hormone, is synthesised in response to various abiotic stresses and regulates the expression of numerous stress-responsive genes in plants [37]. In this study, we found that the expression of eight *OsOSCA* genes was upregulated by at least one type of osmotic-related abiotic stress, such as PEG, NaCl, drought, or ABA treatment; in contrast, the expression of *OsOSCA1.3* was decreased by PEG and ABA treatment. We also found that *OsOSCA2.2* and *OsOSCA2.3* were not regulated by these four kinds of abiotic-related stress. In particular, the expression of *OsOSCA2.2* was only showed as circadian rhythm oscillation, but not in osmotic-related abiotic stress, which indicated that *OsOSCA2.2* plays a different role in sensing and responsing to water potential in guard cells. These results suggested that each member of the *OsOSCA* family plays a distinct role in the growth and development and the responses to diverse abiotic stresses, and provided further clues for the study of the physiological function of OsOSCAs as an osomosensor in rice.

## Conclusions

OSCA was first characterised as an osmosensor that mediated hyperosmolality-induced [Ca^2+^]_i_ increases in *Arabidopsis*, indicating that this multiple-member family may play pivotal roles in sensing the exogenous and endogenous osmotic changes and in regulating plant growth and development. Sequence and phylogenetic analyses showed that 11 OsOSCAs from *Oryza sativa* L. *Japonica* contained a conserved DUF221 domain and shared common structural characteristics with their homologs in *Oryza sativa* L. ssp. *Indica*, *Oryza glaberrima*, and *Oryza brachyantha*. In addition, we demonstrated that the expression of *OsOSCAs* was correlated with various exogenous and endogenous osmotic changes in an organ/tissue-specific manner in rice.

## Methods

### Plant material, growth conditions, and osmotic-related stress treatment

Rice plants (*Oryza sativa* L. spp. *japonica* cv. Zhonghua11) were planted in a growth chamber and in fields (from May to October, annually) at Beijing Normal University (Beijing, China). For growth in the chamber, seeds were incubated for at least 1 week at 42 °C to break any dormancy, and then soaked in water at 20 °C for 3 days and germinated for 1 day at 37 °C. The most uniformly germinated seeds were transferred into a 96-well plate, from which the bottom was removed. The plate was floated on water for 1 day at 37 °C in the dark to promote root growth and then transferred into a growth chamber with a 14-h light (24 °C)/10-h dark (20 °C) photoperiod. Five days later, the seedlings were cultured with Yoshida’s culture medium, which was replaced every 2 days. For osmotic-related stress treatment, 30-day-old seedlings were separately transferred into Yoshida’s culture medium containing 150 mM NaCl, 20 % (w/v) PEG 6000, and 100 μM ABA, with ethyl alcohol (100 μL/L final concentration) as a control. In addition, 30-day-old seedlings were placed in the growth chamber at 50–60 % relative humidity for drought-stress treatment.

For growth in the fields, the rice seeds were soaked in 1 % carbendazim for 1 day and in water at 20 °C for 3–5 days. Then, the most uniformly germinated seeds were sown in the seed bed until approximately the four-true-leaf stage. Seedlings were transplanted into the field and grown for about five months. Field management, including irrigation, fertiliser application, and pest control essentially followed normal agricultural practices. To analyse the expression pattern of *OsOSCA* genes, the following tissues and organs were collected: 30-day-old root (Rt) and shoot (St), mature stem (Sm), mature flag leaf (Fl), stamen (Sn), pistil (Pi), and mature seed (Sd, 45 days after pollination), the different stages of caryopsis development, including 0, 2, 4, 8, 12, 20, and 30 days after pollination, and seed imbibition at 0, 1, 6, 12, 24, 48, and 72 h at room temperature. All materials were collected at the indicated times, immediately frozen in liquid nitrogen, and stored at −80 °C prior to RNA extraction.

### Identification of OSCAs in rice

We used fifteen Arabidopsis OSCAs to perform a TBLASTN search of the rice genome database (http://rice.plantbiology.msu.edu/) and obtained the cDNA and protein sequence of OsOSCA in *Oryza sativa* L. ssp. *Japonica*. OsOSCA orthologues of *Oryza sativa* L. ssp. *Indica* (OsIOSCAs), *Oryza glaberrima* (OgOSCAs), and *Oryza brachyantha* (ObOSCAs) were obtained from Ensembl Genomes (http://www.ensembl.org/index.html) using the best reciprocal BLAST software. The Simple Modular Architecture Research Tool (SMART; http://smart.embl-heidelberg.de/smart/set_mode.cgi?NORMAL=1) was used to identify OSCAs with the presence of DUF221 and other typical domains in their protein structure. The general information and sequence characteristics of 45 OSCAs from four rice species are listed in Additional file 1: Table S1. Gene structures of OSCAs were analysed on the Gene Structure Display Server 2.0 (GSDS; http://gsds.cbi.pku.edu.cn/).

### Phylogenetic analysis and sequence alignment

Full-length CDS sequences of *OSCA* genes from different rice species were aligned using the ClustalX 1.83 software [38] and the phylogenetic tree was constructed using MEGA v5 with the neighbour-joining method [39]. A total of 1,000 bootstrap replicates were performed in each analysis to obtain confidence support. The conserved protein domain of DUF221 in OsOSCAs was analysed using DNAMAN software (http://www.lynnon.com/dnaman.html) and modified manually [40]. The TM domains in OsOSCAs were annotated according to TMHMM Server v. 2.0 predictions (http://www.cbs.dtu.dk/services/TMHMM/).

### RNA extraction and quantitative real-time PCR

Total RNA was isolated from rice tissues using the TRIzol® reagent (Invitrogen, USA) and purified using a PureLink® RNA Mini Kit (Invitrogen) combined with the PureLink® DNase kit (Invitrogen), according to the manufacturer’s protocol. Approximately 4 μg of total RNA was reverse-transcribed using the Reverse-Aid™ First Strand cDNA Synthesis Kit to generate cDNA (Fermentas, Canada). Quantitative real-time PCR (qRT-PCR) was performed on the 7500 Fast Real-Time PCR System (Applied Biosystems, USA) using Power SYBR® Green PCR Master Mix (Applied Biosystems). The thermal program was 2 min at 50 °C, 10 min at 95 °C, followed by 40 cycles of 15 s at 95 °C and 60 s at 60 °C. The data were normalised to the rice *actin* gene (LOC_Os03g61970.1) [41] using the ΔΔCT method, as described previously [42]. To investigate whether the expression of actin is stability under different abiotic stresses, the expression of other two housekeeping gene *eEF1a* (LOC_Os03g08020) and *UBQ5* (LOC_Os01g22490) was detected compared to *actin*. Gene expression stability values (M) of these three genes were calculated using geNorm as described by Vandesompele et al. [43]. The dissociation curve program was used to confirm the specificity of the target amplification product. All primers used in this study are listed in Additional file 2: Table S2. At least three independent biological replicates were performed for qRT-PCR analysis. Value changes of more than twofold (>2 or <0.5) were considered to indicate the induction or repression of *OsOSCA* expression. Analysis was performed using the Data Processing System, and a one-way analysis of variance (ANOVA) and Tukey’s multiple range test [44] were conducted to determine significant differences. *P* < 0.05 was considered to indicate statistical significance.

### In silico expression analysis of OsOSCAs

OsOSCA gene microarray data were extracted from the Rice Functional Genomic Expression Database (http://signal.salk.edu/cgi-bin/RiceGE) to analyse the expression profiles of OsOSCAs in organs during different developmental stages (GSE6893) (Additional file 3: Table S3). The absolute signal values were respectively divided by the average of all absolute values.

### Availability of supporting data

All relevant supporting data can be found within the supplementary files accompanying this article. Phylogenetic data supporting the results of this article are available in the TreeBASE repository, http://purl.org/phylo/treebase/phylows/study/TB2:S17910.

## Additional files

Additional file 1: Table S1. General information and sequence characterisation of 45 *OSCA* genes from *Oryza sativa* L. ssp. *Japonica*, *Oryza sativa* L. ssp. *Indica*, *Oryza glaberrima*, and *Oryza brachyantha*. (DOC 89 kb) Additional file 2: Table S2. Sequences of oligonucleotide primers for qRT-PCR. F: forward; R: reverse. (DOC 52 kb) Additional file 3: Table S3. Microarray data of *OsOSCA* expression patterns during panicle growth and caryopsis development in rice. (DOC 40 kb) Additional file 4: Table S4. The relative expression of three housekeeping genes: *actin*, *eEF1a* and *UBQ5* at different abiotic-related stress treatment condition was detected by qRT-PCR. (DOC 25 kb) Additional file 5: Figure S1. Predicted conserved domains in OSCAs of *Oryza sativa* L. ssp. *Japonica*, *Oryza sativa* L. ssp. *Indica*, *Oryza glaberrima*, and *Oryza brachyantha*. (DOC 233 kb) Additional file 6: Figure S2. Relative water content of rice caryopsis at different stages after pollination. (DOC 41 kb) Additional file 7: Figure S3. The relative water content of ZH11 seeds during imbibition in solutions containing various PEG concentrations. (DOC 53 kb) Additional file 8: Figure S4. Circadian rhythmic expression of the marker gene *OsLHY* in four-leaf-stage ZH11 seedlings. (DOC 56 kb) Additional file 9: Figure S5. Expression of marker genes in roots of four-leaf-stage rice in the presence of osmotic-related abiotic stresses. (DOC 67 kb)

## Abbreviations

DAF: days after flowering
HAI: hours after imbibition
OICI: hyperosmolality-induced [Ca^2+^]_i_ increases
OSCA: hyperosmolality-gated calcium-permeable channels
