# Supplementary material for: Genome-wide survey and expression analysis of the OSCA gene family in rice
Source: BMC Plant Biol. 2015 Oct 26;15:261. doi: 10.1186/s12870-015-0653-8 (PMC4624379; doi:10.1186/s12870-015-0653-8)
Supplement: Additional file 1: Table S1. — General information and sequence characterisation of 45 OSCA genes from Oryza sativa L. ssp. Japonica, Oryza sativa L. ssp. Indica, Oryza glaberrima, and Oryza brachyantha. (DOC 89 kb) [file 12870_2015_653_MOESM1_ESM.doc]

**Table S1. General information and sequence characterisation of 45 *OSCA* genes from *Oryza sativa* L. ssp. *Japonica*, *Oryza sativa* L. ssp. *Indica*, *Oryza glaberrima*, and *Oryza brachyantha*.**

| Genes | Gene ID | ORF (bp) | Protein Size (aa) | TM |
| --- | --- | --- | --- | --- |
| ***OsOSCA1.1*** | LOC_Os01g35050 | 2307 | 768 | 9 |
| ***OsOSCA1.2*** | LOC_Os05g51630 | 2301 | 766 | 9 |
| ***OsOSCA1.3*** | LOC_Os05g32720 | 2304 | 767 | 8 |
| ***OsOSCA1.4*** | LOC_Os10g42820 | 2433 | 810 | 8 |
| ***OsOSCA2.1*** | LOC_Os12g43720 | 2292 | 763 | 9 |
| ***OsOSCA2.2*** | LOC_Os03g47070 | 1443 | 480 | 6 |
| ***OsOSCA2.3*** | LOC_Os03g51620 | 2232 | 743 | 10 |
| ***OsOSCA2.4*** | LOC_Os12g39320 | 2088 | 695 | 9 |
| ***OsOSCA2.5*** | LOC_Os01g72210 | 2106 | 701 | 7 |
| ***OsOSCA3.1*** | LOC_Os07g05570 | 2196 | 731 | 10 |
| ***OsOSCA4.1*** | LOC_Os03g04450 | 2379 | 792 | 9 |
|  |  |  |  |  |
| ***OsIOSCA1.1*** | BGIOSGA001480-PA | 2307 | 768 | 9 |
| ***OsIOSCA1.2*** | BGIOSGA020484-PA | 2301 | 766 | 9 |
| ***OsIOSCA1.3*** | BGIOSGA019816-PA | 2352 | 783 | 8 |
| ***OsIOSCA1.4*** | BGIOSGA031326-PA | 2436 | 811 | 8 |
| ***OsIOSCA2.1*** | BGIOSGA001873-PA | 2292 | 763 | 9 |
| ***OsIOSCA2.2*** | BGIOSGA010070-PA | 2334 | 777 | 9 |
| ***OsIOSCA2.3*** | BGIOSGA009887-PA | 2232 | 743 | 10 |
| ***OsIOSCA2.4*** | BGIOSGA037680-PA | 2088 | 695 | 9 |
| ***OsIOSCA2.5*** | BGIOSGA005174-PA | 2040 | 679 | 7 |
| ***OsIOSCA3.1*** | BGIOSGA024816-PA | 2196 | 731 | 10 |
| ***OsIOSCA4.1*** | BGIOSGA011808-PA | 2379 | 792 | 9 |
|  |  |  |  |  |
| ***ObOSCA1.1*** | OB01G28470.1 | 2310 | 769 | 9 |
| ***ObOSCA1.2*** | OB05G35570.1 | 2313 | 770 | 9 |
| ***ObOSCA1.3*** | OB05G23290.1 | 2304 | 767 | 8 |
| ***ObOSCA1.4*** | OB10G26890.1 | 2424 | 807 | 8 |
| ***ObOSCA2.1*** | OB12G26520.1 | 2295 | 764 | 9 |
| ***ObOSCA2.2*** | OB03G36630.1 | 2340 | 779 | 10 |
| ***ObOSCA2.3*** | OB03G39680.1 | 2232 | 743 | 10 |
| ***ObOSCA2.4*** | OB12G24020.1 | 2097 | 698 | 8 |
| ***ObOSCA2.5*** | OB01G53150.1 | 2145 | 714 | 9 |
| ***ObOSCA3.1*** | OB07G12430.1 | 2190 | 729 | 10 |
| ***ObOSCA4.1*** | OB03G12950.1 | 1908 | 635 | 6 |
|  |  |  |  |  |
| ***OgOSCA1.1*** | ORGLA01G0152400.1 | 2307 | 768 | 9 |
| ***OgOSCA1.2*** | ORGLA05G0244400.1 | 2301 | 766 | 9 |
| ***OgOSCA1.3*** | ORGLA05G0132500.1 | 2304 | 767 | 8 |
| ***OgOSCA1.4*** | ORGLA10G0149500.1 | 2433 | 810 | 8 |
| ***OgOSCA2.1*** | ORGLA12G0171200.1 | 2292 | 763 | 9 |
| ***OgOSCA2.2*** | ORGLA03G0295500.1 | 2334 | 777 | 9 |
| ***OgOSCA2.3*** | ORGLA03G0295500.1 | 2232 | 743 | 10 |
| ***OgOSCA2.4*** | ORGLA12G0171200.1 | 2088 | 695 | 9 |
| ***OgOSCA2.5*** | ORGLA01G0378500.1 | 2124 | 707 | 9 |
| ***OgOSCA3.1*** | ORGLA07G0027500.1 | 2196 | 731 | 10 |
| ***OgOSCA4.1_1*** | ORGLA03G0027400.1 | 2379 | 792 | 9 |
| ***OgOSCA4.1_2*** | ORGLA12G0183200.1 | 2379 | 792 | 9 |
