# Supplementary material for: Genome-wide survey and expression analysis of the OSCA gene family in rice
Source: BMC Plant Biol. 2015 Oct 26;15:261. doi: 10.1186/s12870-015-0653-8 (PMC4624379; doi:10.1186/s12870-015-0653-8)
Supplement: Additional file 2: Table S2. — Sequences of oligonucleotide primers for qRT-PCR. F: forward; R: reverse. (DOC 52 kb) [file 12870_2015_653_MOESM2_ESM.doc]

**Table S2. Sequences of oligonucleotide primers for qRT-PCR**. F: forward; R: reverse.

| Oligos | Primer Sequence(5'-3') |
| --- | --- |
| OsOSCA1.1 F | GTGGCAACAATGCGTCT |
| OsOSCA1.1 R | ACAACCTGGTGCGTAA |
| OsOSCA1.2 F | GCTTGTTCTGTTCCCTGTGA |
| OsOSCA1.2 R | CTGTAAACTGGTCTGGTCGC |
| OsOSCA1.3 F | TTTGCTGGCATCGACGAG |
| OsOSCA1.3 R | TGGAATTGAACTTGCTATCTACTG |
| OsOSCA1.4 F | TTTCAAGCCATTCAACATC |
| OsOSCA1.4 R | TGCCTTTCAAACTTCAGC |
| OsOSCA2.1 F | GTCCTTGTTCGTGGAATACC |
| OsOSCA2.1 R | GCCTTCTTAGCACCAGTCAT |
| OsOSCA2.2 F | CGATGTAAATCCGCAAACTG |
| OsOSCA2.2 R | CACAAGTTGCTCAGGCACTAG |
| OsOSCA2.3 F | GCCTCACAACTATGGCGTCT |
| OsOSCA2.3 R | CCAAGCCAGCAACCCGTC |
| OsOSCA2.4 F | TGTCTGTGAGTGATGCTGTTGA |
| OsOSCA2.4 R | GGAGTTTGCCAGTTTGATGA |
| OsOSCA2.5 F | TCACATACATTTTGACCGAC |
| OsOSCA2.5 R | ACAGCATAGACCAACCCA |
| OsOSCA3.1 F | ATTTCCGAGCACTTCATCCTG |
| OsOSCA3.1 R | CCTGTTGTTTTAGACTCCGCATA |
| OsOSCA4.1 F | CAGTGCTGTCTTACTTCTCC |
| OsOSCA4.1 R | TTTCAAGCGATGATTCCA |
| actin F | AGTGGTCGTACAACAGGTA |
| actin R | TCTTCATTAGGCAGTCAGT |
| ABI5 F | GCGGCAGAGGCGGATGAT |
| ABI5 R | GTTCGTCGGAGGCAAAATCT |
| DSM2 F | ATCGCCAACGTGCCCTACT |
| DSM2 R | GCACACCTTCGAACTTGTCCAT |
| OsLHY F | GGGTCGTCTGGCTTTTGAT |
| OsLHY R | CGGTACCCTGTTCTCCTTC |
| OsP5C F | GCTGACATGGATATGGCAAAAC |
| OsP5C R | GTAAGGTCTCCATTGCATTGCA |
| peroxidase 24 precursor F | AACCCATCCTACGCCAGA |
| peroxidase 24 precursor R | CGCCTTGAGGTTCACGAAGTA |
| OsUBQ5 F | ACCACTTCGACCGCCACTACT |
| OsUBQ5 R | ACGCCTAAGCCTGCTGGTT |
| OseEF1α F | TTTCACTCTTGGTGTGAAGCAGAT |
| OseEF1α R | GACTTCCTTCACGATTTCATCGTAA |
