# Supplementary material for: Genome-wide survey and expression analysis of the OSCA gene family in rice
Source: BMC Plant Biol. 2015 Oct 26;15:261. doi: 10.1186/s12870-015-0653-8 (PMC4624379; doi:10.1186/s12870-015-0653-8)
Supplement: Additional file 3: Table S3. — Microarray data of OsOSCA expression patterns during panicle growth and caryopsis development in rice. (DOC 40 kb) [file 12870_2015_653_MOESM3_ESM.doc]

**Table S3. Microarray data of *OsOSCAs* expression patterns during panicle growth and caryopsis development in rice.**

| **Genes** | **P1** | **P2** | **P3** | **P4** | **P5** | **P6** | **S1** | **S2** | **S3** | **S4** | **S5** |
| --- | --- | --- | --- | --- | --- | --- | --- | --- | --- | --- | --- |
| *OsOSCA1.1* | 2.40 | 1.41 | 2.02 | 2.40 | 1.49 | 1.60 | 0.76 | 0.73 | 0.79 | 0.86 | 1.32 |
| *OsOSCA1.2* | 0.43 | -0.21 | -0.05 | 0.50 | -1.02 | 0.59 | 0.74 | -0.56 | -0.49 | -0.61 | -0.18 |
| *OsOSCA1.3* | -6.63 | -5.42 | -5.71 | -6.61 | -6.48 | -4.42 | -6.74 | -8.52 | -6.28 | -4.41 | -5.81 |
| *OsOSCA1.4* | -1.41 | -1.27 | -1.02 | -0.98 | -1.10 | -1.71 | -1.79 | -0.72 | -0.27 | -0.62 | -0.90 |
| *OsOSCA2.1* | -1.53 | -1.88 | -1.55 | -2.03 | -1.67 | -1.43 | -2.00 | -2.64 | -2.73 | -3.05 | -3.19 |
| *OsOSCA2.2* | -0.96 | -0.86 | -1.05 | -1.64 | -1.16 | -1.14 | -1.31 | -1.66 | -1.76 | -2.60 | -2.22 |
| *OsOSCA2.3* | -5.42 | -5.58 | -4.76 | -5.42 | -6.94 | -3.28 | -6.03 | -2.82 | -3.07 | -3.55 | -5.84 |
| *OsOSCA2.4* | 0.38 | 0.28 | 0.05 | 0.05 | -0.18 | 0.10 | 0.20 | 1.30 | 1.64 | 1.40 | 1.21 |
| *OsOSCA2.5* | -2.36 | -3.03 | -3.08 | -3.53 | -3.53 | -3.66 | -3.44 | -2.23 | -1.09 | 0.45 | 1.38 |
| *OsOSCA3.1* | 1.67 | 1.51 | 1.32 | 0.97 | 0.43 | 1.31 | 0.82 | 0.14 | 0.48 | 0.67 | 0.89 |
| *OsOSCA4.1* | -0.16 | -0.51 | -0.42 | -0.76 | -0.59 | -0.81 | -0.51 | 0.03 | 0.64 | 1.51 | 1.59 |
