# Supplementary material for: Genome-wide survey and expression analysis of the OSCA gene family in rice
Source: BMC Plant Biol. 2015 Oct 26;15:261. doi: 10.1186/s12870-015-0653-8 (PMC4624379; doi:10.1186/s12870-015-0653-8)
Supplement: Additional file 4: Table S4. — The relative expression of three housekeeping genes: actin, eEF1a and UBQ5 at different abiotic-related stress treatment condition was detected by qRT-PCR. (DOC 25 kb) [file 12870_2015_653_MOESM4_ESM.doc]

**Table S4.** The relative expression of three housekeeping genes: actin, eEF1a and UBQ5 at different abiotic-related stress treatment condition was detected by qRT-PCR.
