# Supplementary figures and images for: Genome-wide survey and expression analysis of the OSCA gene family in rice
Source: BMC Plant Biol. 2015 Oct 26;15:261. doi: 10.1186/s12870-015-0653-8 (PMC4624379; doi:10.1186/s12870-015-0653-8)

**Figure S1**


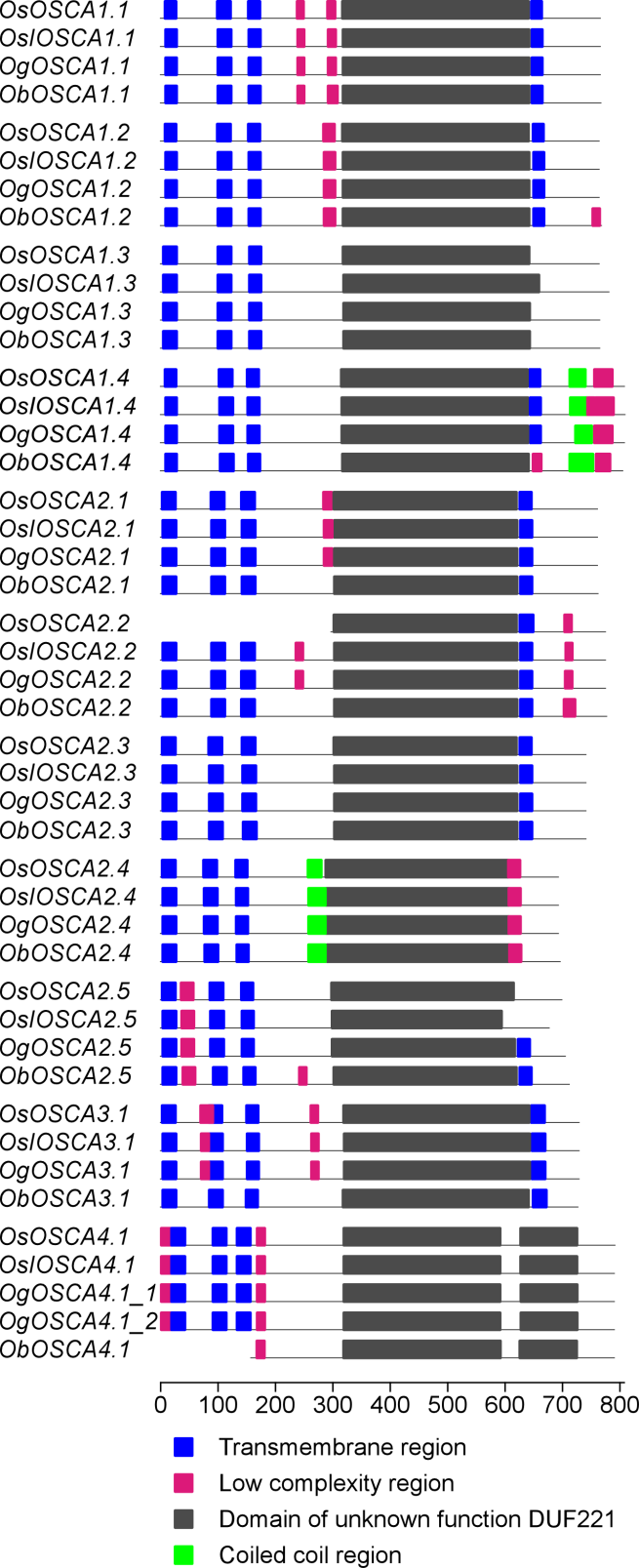

Supplement: Additional file 5: Figure S1. — Predicted conserved domains in OSCAs of Oryza sativa L. ssp. Japonica, Oryza sativa L. ssp. Indica, Oryza glaberrima, and Oryza brachyantha. (DOC 233 kb) [file 12870_2015_653_MOESM5_ESM.doc]

**Figure S2**

**
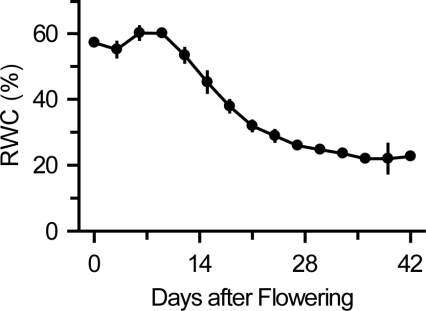
**

Supplement: Additional file 6: Figure S2. — Relative water content of rice caryopsis at different stages after pollination. (DOC 41 kb) [file 12870_2015_653_MOESM6_ESM.doc]

**Figure S3**

**
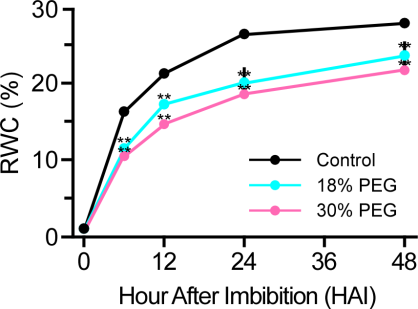
**

Supplement: Additional file 7: Figure S3. — The relative water content of ZH11 seeds during imbibition in solutions containing various PEG concentrations. (DOC 53 kb) [file 12870_2015_653_MOESM7_ESM.doc]

**Figure S4**


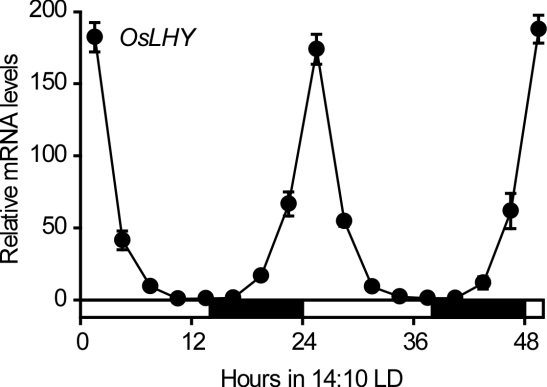

Supplement: Additional file 8: Figure S4. — Circadian rhythmic expression of the marker gene OsLHY in four-leaf-stage ZH11 seedlings. (DOC 56 kb) [file 12870_2015_653_MOESM8_ESM.doc]

**Figure S5**

**
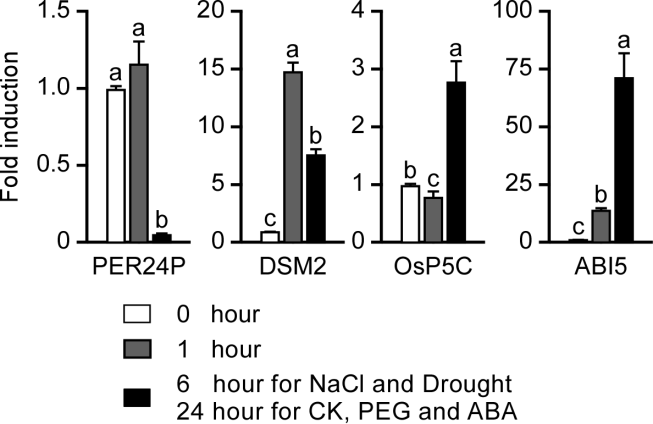
**

Supplement: Additional file 9: Figure S5. — Expression of marker genes in roots of four-leaf-stage rice in the presence of osmotic-related abiotic stresses. (DOC 67 kb) [file 12870_2015_653_MOESM9_ESM.doc]
